# Supplementary material for: Identification of ER/SR resident proteins as biomarkers for ER/SR calcium depletion in skeletal muscle cells
Source: Orphanet J Rare Dis. 2022 Jun 13;17:225. doi: 10.1186/s13023-022-02368-9 (PMC9195201; doi:10.1186/s13023-022-02368-9)
Supplement: Supplementary file 1 — Additional file 1. Figure S1 Identification of extracellular ERS proteins. A) Blue stain gel of concentrated media from skeletal muscle cells treated with vehicle or 100nM Tg for 8 hours. Arrows indicate qualitative protein increases. B) Table of extracellular ERS proteins identified by mass spectrometry, UniProt accession number, and average Tg-induced response, p-values listed, 2-tailed t-test, vehicle normalized abundance vs Tg normalized abundance. [file 13023_2022_2368_MOESM1_ESM.pdf]

Supplemental Figure 1

A

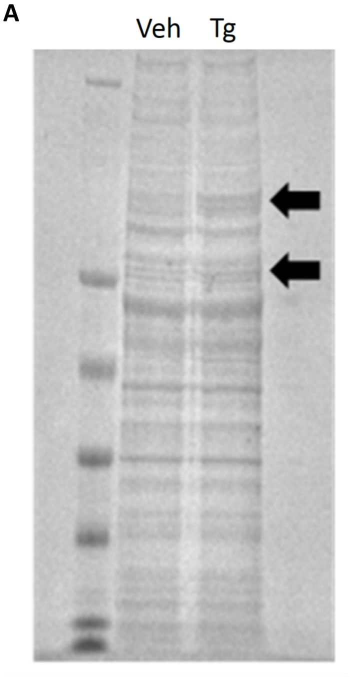

B

UniProt Accession Number	Abbreviation	Average Tg-Induced Response	p-value
Q16270	IGFBP7	0.52625	0.061153322
Q8WUF8	C5orf21	0.66075	0.002348763
Q99574	SERPINI1	0.76375	0.147792986
P51688	SGSH	0.78175	0.083365481
Q12860	CNTN1	0.793	0.136250777
Q96D15	RCN3	0.8515	0.090516444
Q9Y680	PDIA6	0.96075	0.303903433
Q9NWM8	FKBP14	0.96375	0.420473796
Q13438	OS9	1.1595	0.816982272
Q8NBJ7	SUMF2	1.1865	0.170210893
P50454	SERPINH1	1.24525	0.092832136
O00461	GOLPH4	1.26	0.015599482
Q14257	RCN2	1.2815	0.45938516
O43852	CALU	1.645	0.120681603
Q14554	PDIA5	1.6885	0.042730624
Q8IXB1	DNAJC10	1.95125	0.038111254
Q9UNW1	MINPP1	1.994	0.000676464
O95302	FKBP9	2.20075	0.064938688
Q15293	RCN1	2.21575	0.116923614
O00748	CES2	2.37225	0.05965484
P26885	FKBP2	2.741	0.056324401
Q9H488	POFUT1	2.884	0.09720499
Q9BZQ6	EDEM3	3.1905	0.039869986
P13667	PDIA4	3.34375	0.043085599
Q6UW63	KDELC1	3.34525	0.069823548
P30533	LRPAP1	3.59525	0.054476796
Q32P28	LEPRE1	3.653	0.028528902
Q9BS26	ERP44	3.73925	0.082017722
P55145	MANF	3.828	0.01713504
Q9HCN8	CALR	3.8725	0.048518366
Q99470	SDF2	3.92775	0.033723365
P30101	PDIA3	4.0315	0.025813568
Q6Y288	B3GALTL	4.03375	0.049119636
P07237	P4HB	4.1	0.008270035
Q8NBS9	TXNDC5	4.2215	0.047682763
Q9BT09	TNRC5	4.247	0.025407213
Q14696	MESDC2	4.423	0.026045815
Q969H8	MYDFG	4.61625	0.011364641
P30040	ERP29	4.823	0.019520103
Q6UXH1	CRELD2	4.844	0.001570714
P27797	KTELC1	4.942	0.021529048
Q7Z4H8	KDELC2	5.0625	0.045153687
Q8IVL5	LEPREL1	5.12825	0.017258837
Q9UMX5	NENF	5.18575	0.011169654
Q5NDL2	AER61	5.4185	0.041745397
P11021	HSPA5	5.6115	0.035165814
Q9NYU2	UGCGL1	6.0175	0.014082415
Q8IWF2	FOXRED2	6.07975	0.00472411
Q8IYK4	FKBP7	6.3025	0.006959466
Q96AY3	GLT25D2	6.39375	0.023323587
P14625	HSP90B1	6.45475	0.009122763
Q8IVL6	LEPREL2	6.71325	0.019982992
Q15084	PCYOX1	6.963	0.024711408
Q8N129	CNPY4	7.75625	0.014727488
Q9Y4L1	HYOU1	28.555	0.068748742
