# Supplementary figures and images for: Identification of ER/SR resident proteins as biomarkers for ER/SR calcium depletion in skeletal muscle cells
Source: Orphanet J Rare Dis. 2022 Jun 13;17:225. doi: 10.1186/s13023-022-02368-9 (PMC9195201; doi:10.1186/s13023-022-02368-9)

Supplementary Figure 2

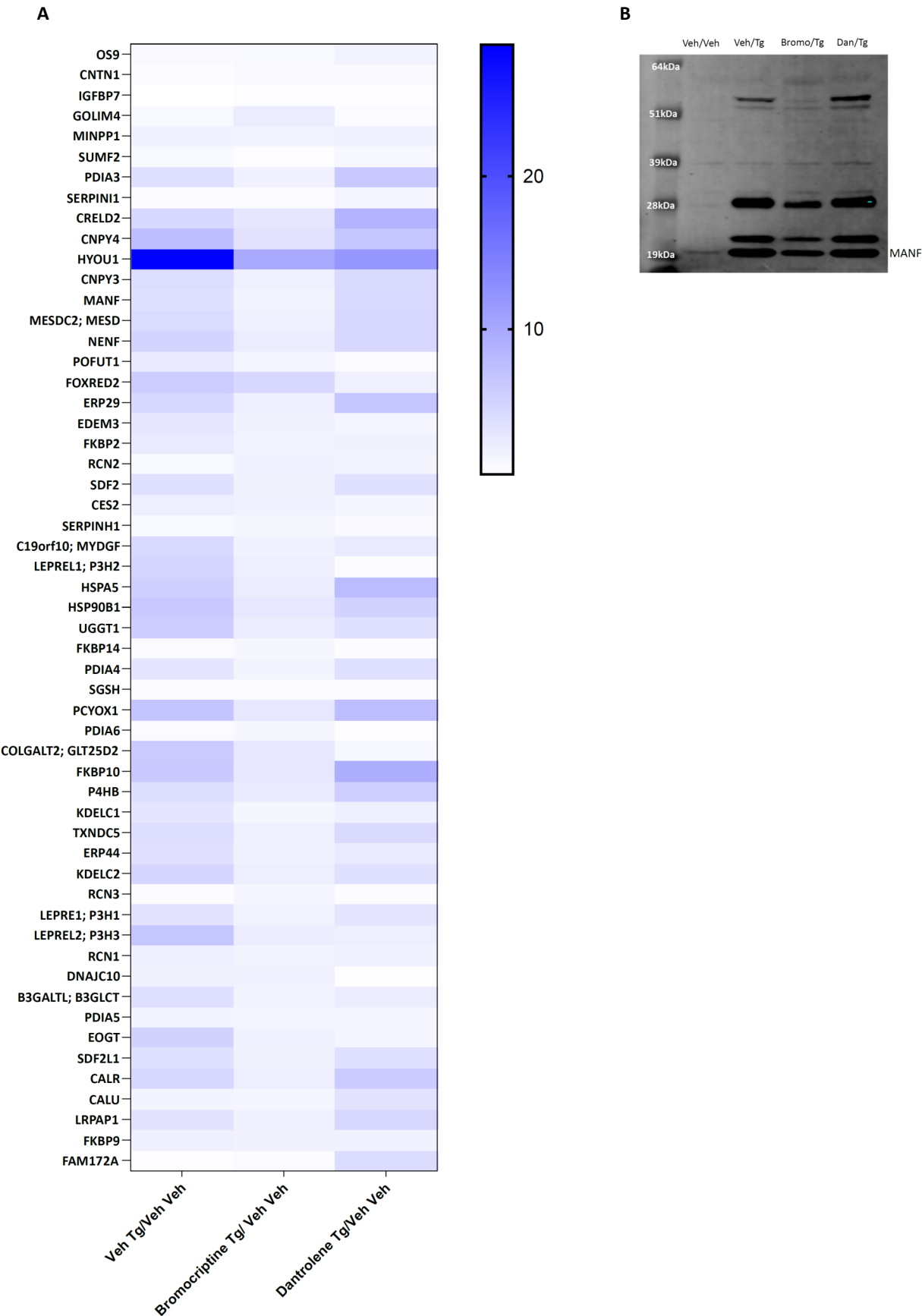

Supplement: Supplementary file 2 — Additional file 2. Figure S2 Extracellular ERS proteins pre-treated with bromocriptine or dantrolene A) Heat map depicting changes in extracellular ERS protein content when pre-treated with 20µM bromocriptine, or 50µM dantrolene 30 minutes prior to 100nM Tg for 8 hours. B) Western blot of concentrated media collected from T0034 skeletal muscle cell line pre-treated with vehicle, 20µM bromocriptine, or 50µM dantrolene, mean ± SEM, n=2/treatment groups. [file 13023_2022_2368_MOESM2_ESM.pdf]

Supplemental Figure 3

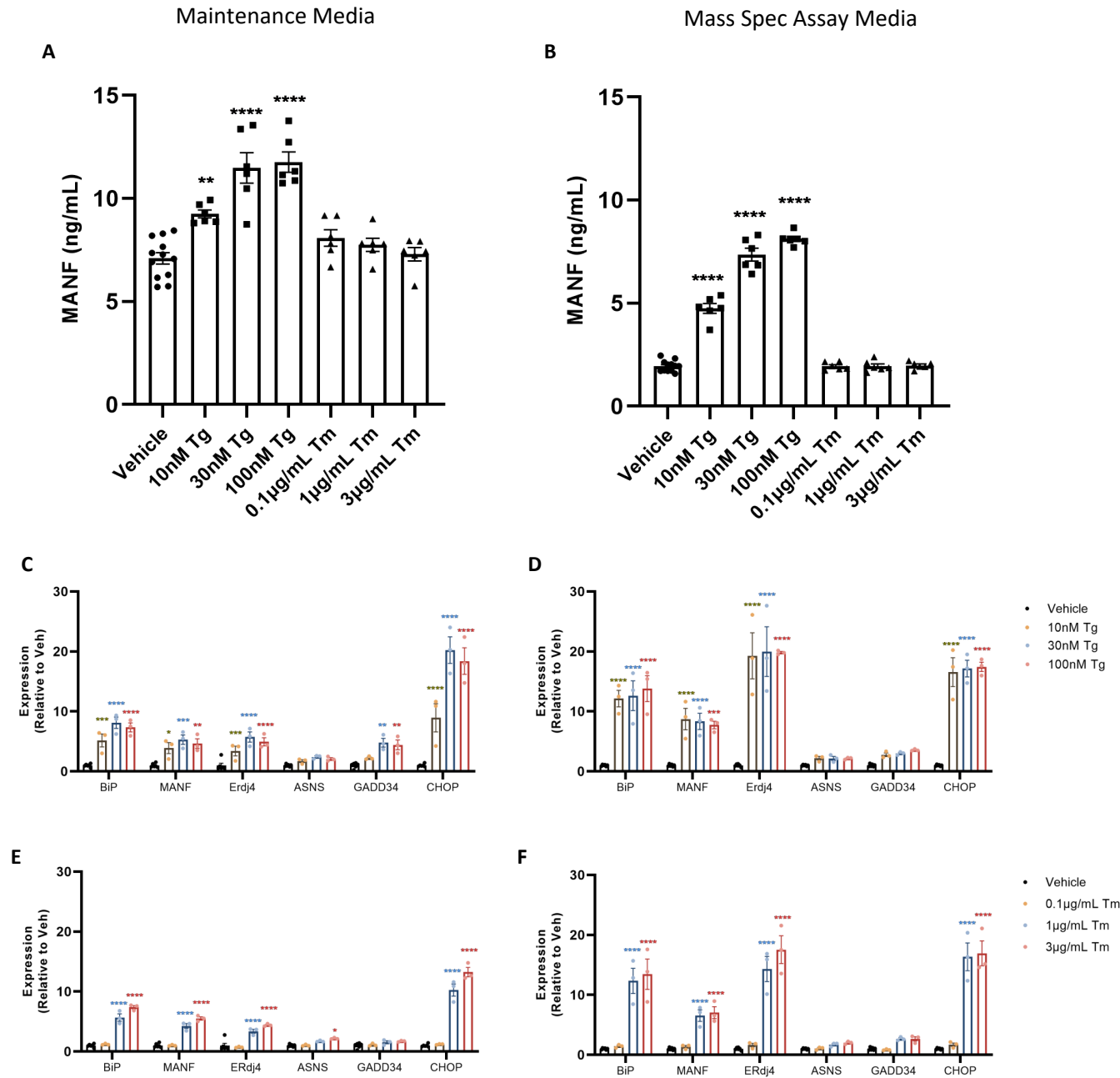

Supplement: Supplementary file 3 — Additional file 3. Figure S3 Exodosis is triggered by Tg-induced ER/SR calcium depletion but not Tm-induced ER stress A, B) MANF HTRF assay of media collected from T0034 skeletal muscle cell line treated with Tg or Tm in maintenance media (A) or mass spectrometry assay media (B) for 8 hours. Extracellular MANF is exclusively elevated following Tg treatment, mean ± SEM, n= 6-12 wells/treatment group, **p<0.01, ****p<0.0001, 1-way ANOVA, Dunnett’s multiple comparison test, Tg or Tm vs vehicle. C-F) UPR and ER stress response mRNA levels from T0034 skeletal muscle cell line treated with Tg or Tm in maintenance media (C and E, respectively) or mass spectrometry assay media (D and F, respectively) for 8 hours. Activation of UPR and ER stress response is not sufficient to trigger exodosis, mean ± SEM, n= 3 wells/treatment group, *p<0.05, **p<0.01, ***p<0.001, ****p<0.0001, 2-way ANOVA, Dunnett’s multiple comparison test, Tg or Tm vs vehicle. [file 13023_2022_2368_MOESM3_ESM.pdf]
